# Subcutaneous Emphysema in Spontaneous Acute Colonic Rupture Mimicking an Acute Traumatic Injury

**DOI:** 10.7759/cureus.80701

**Published:** 2025-03-17

**Authors:** Uyen Nguyen, Kailey Ziemianski, Michael Galuska

**Affiliations:** 1 Emergency Department, AppolloMD, Brooklyn, USA; 2 Emergency Department, US Acute Care Solutions/Allegheny Health Network (USACS/AHN), Pittsburgh, USA; 3 Emergency Medicine, Duke Lifepoint Conemaugh Memorial Medical Center, Johnstown, USA

**Keywords:** acute colonic perforation, case report, mimic, mimic disease, subcutaneous emphysema, trauma

## Abstract

Subcutaneous emphysema often results from traumatic injuries such as pneumothorax or traumatic perforation of the bowel. In this case report, however, the authors present subcutaneous emphysema that resulted from a spontaneous colonic perforation that disguised itself as a traumatic injury in an elderly patient who was presumed to have sustained a traumatic injury.

In this case, an 88-year-old male presented to the emergency department via trauma alert after being found unresponsive on the floor with extensive subcutaneous emphysema of his hips, thighs, chest, abdomen, and cervical area. He was intubated prior to arrival and had bilateral tube thoracostomies placed shortly after arrival. After stabilization, computed tomography (CT) of the head, cervical spine, chest, abdomen, and pelvis was performed, and he was found to have an acute colonic perforation, which resulted in extensive subcutaneous emphysema that mimicked the presumed traumatic injury on presentation.

This case highlights the importance of keeping a broad differential diagnosis in patients with extensive subcutaneous emphysema. In this case, the patient presented with a presumed acute traumatic injury, though he was ultimately diagnosed with acute colonic perforation.

## Introduction

Subcutaneous emphysema can result from a multitude of causes, including traumatic, infectious, or spontaneous etiologies. However, in cases of trauma, subcutaneous emphysema is most commonly associated with the thoracic or cervical regions, secondary to injuries involving the thoracic cavity, sinus cavities, or facial bones [1]. Therefore, a spontaneous colonic perforation causing subcutaneous emphysema that mimics a traumatic injury to an extremity is rather unusual. In this case report, the authors present a patient who developed extensive subcutaneous emphysema in the cervical, mediastinal, retroperitoneal, and lower extremity regions due to a spontaneous acute colonic perforation.

## Case presentation

**Figure 1:**
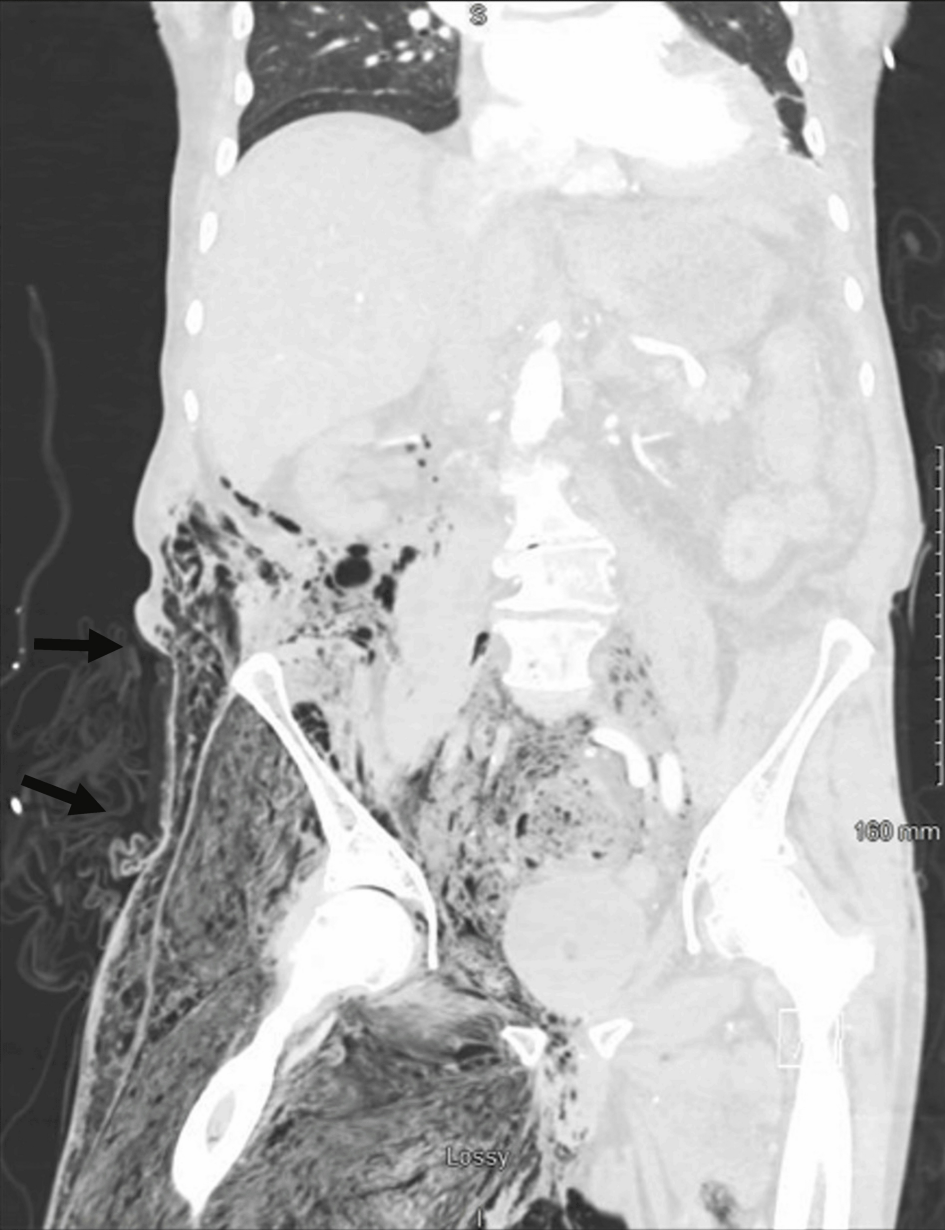
Extensive subcutaneous emphysema in the medial and lateral aspects of the right thigh. Air travels to the anterior compartment of the thigh wall, infiltrating the myofascial plane and causing expansion of the thigh's size.

**Figure 2:**
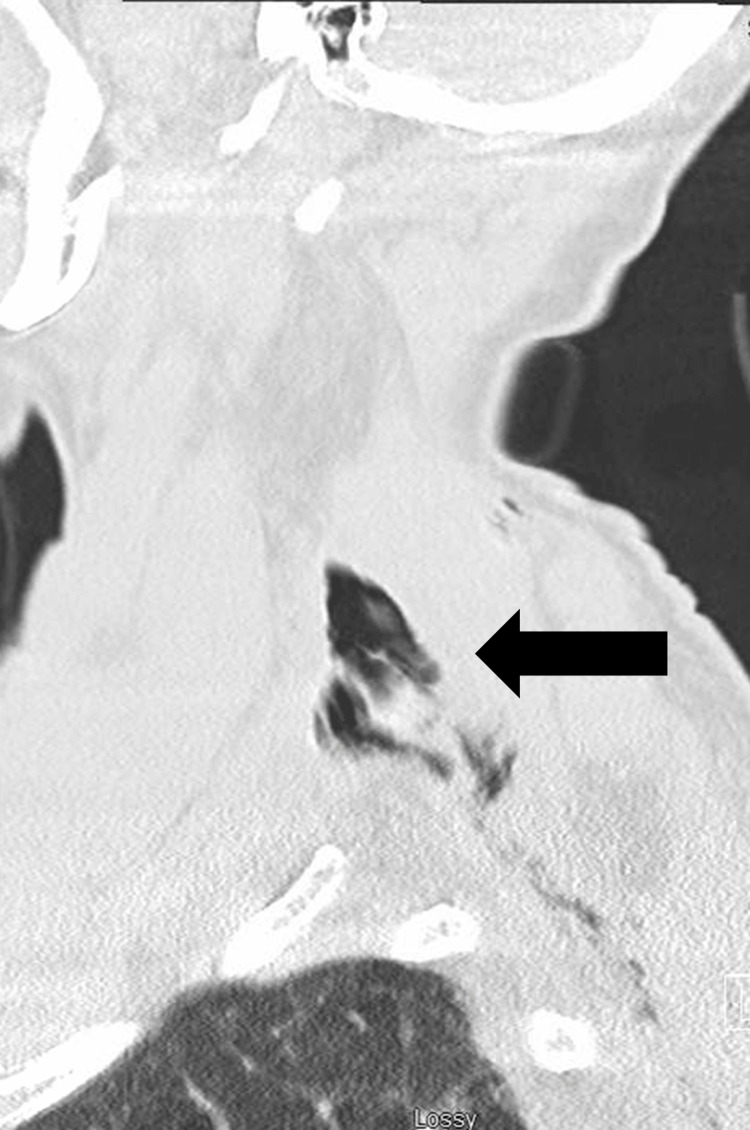
Sagittal view of CT cervical spine reveals subcutaneous emphysema tracking from the posterior thoracic region to the neck, above the apex of the lung. CT: computed tomography

**Figure 3:**
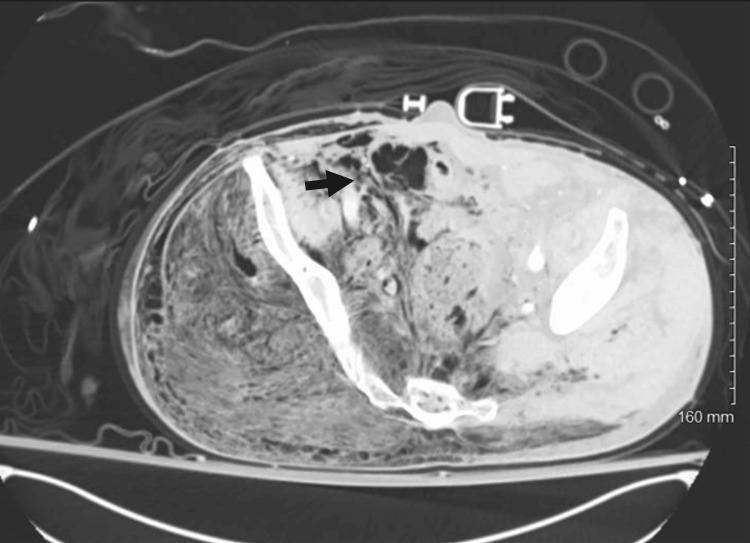
Subcutaneous air within the intra-abdominal content and the right-sided retroperitoneal region. Acute rupture of the ascending colon causes retroperitoneal emphysema, with accumulated air extending into the pelvic wall and pushing the rectum to the left side.

An 88-year-old male was found unresponsive on the floor of his home by a neighbor, with an unknown last known well. The patient was found to be hypotensive, with concerns for a pelvic fracture based on prehospital findings of significant hematoma, swelling, and crepitus in the right hip and chest. A pelvic binder was placed during prehospital care, and packed red blood cells and tranexamic acid were administered for what was expected to be secondary to hemorrhagic shock. Emergency medical services (EMS) personnel also intubated the patient prior to arrival. The patient was presumed to have had multiple traumatic injuries from the suspected fall and was transported directly to the trauma bay upon arrival. His presenting vital signs included a heart rate of 116 beats/min, a blood pressure of 85/68 mmHg, and a peripheral oxygen saturation (SpO_2_) of 99% on 100% fraction of inspired oxygen (FiO_2_). On initial evaluation in the trauma bay, the patient was found to have crepitus and swelling of the right lower extremity, as well as ecchymosis on the right hip. The patient also had evidence of crepitus extending to the right chest wall. Bilateral thoracostomies were placed during resuscitation for a possible tension pneumothorax, given his unresponsive hypotension. The massive transfusion protocol was initiated, and his hypotension improved following these interventions. A standard trauma laboratory panel was obtained during resuscitation (Table 1). After the patient was stabilized, he underwent computed tomography (CT) imaging of his head, cervical spine, chest, abdomen, and pelvis. CT images showed extensive subcutaneous emphysema in the right cervical, mediastinum, retroperitoneum, and right lower extremity, most prominently in the gluteus area and posterior thigh, as evidenced by Figures 1-3. The CT scan demonstrated that the extensive pneumoperitoneum originated from an ascending colonic rupture, with no other acute traumatic injuries identified on imaging. The patient was taken emergently to the operating room for a right hemicolectomy and washout. Despite multiple efforts to resuscitate the patient in the postoperative period, he remained in septic shock while on maximum doses of norepinephrine and vasopressin. His family ultimately decided to transition the patient to comfort care, and he passed away shortly afterward.

**Table 1:** Trauma panel laboratory values CO_2_: carbon dioxide; pCO_2_: partial pressure of carbon dioxide; pO_2_: partial pressure of oxygen; HCO_3_: bicarbonate

Test	Component	Result	Units	Normal range
Complete blood count (CBC)	White blood cells (WBC)	1.5	10³/µL	4.5-11.0
	Hemoglobin (Hgb)	9.8	g/dL	14-18
	Hematocrit (Hct)	30	%	40-54
	Platelets	113	10³/µL	140-440
Basic metabolic panel (BMP)	Sodium (Na)	137	mmol/L	136-145
	Potassium (K)	4.5	mmol/L	3.5-5.1
	Chloride (Cl)	109	mmol/L	98-107
	CO_2_	12	mmol/L	22-29
	Blood urea nitrogen (BUN)	40	mg/dL	9-21
	Creatinine (Cr)	1.6	mg/dL	0.6-1.1
	Glucose	134	mg/dL	70-105
Creatine kinase (CK)	Total CK	6,770	U/L	30-280
Coagulation	Prothrombin time (PT)	19.3	sec	9-12
	International normalized ratio (INR)	1.8		0.9-1.1
	Partial thromboplastin time (PTT)	55	sec	23-35
Arterial blood gas (ABG)	pH	7.31		7.35-7.25
	pCO_2_	26	mmHg	36-46
	pO_2_	289	mmHg	70-102
	HCO_3_	13	meq/L	22-26
	Lactate	8.2	mmol/L	0.5-2.0

## Discussion

Subcutaneous emphysema is defined as air in the subcutaneous tissue and can result from a multitude of pathologies in any location of the body, presenting as crepitus on palpation. Most commonly, subcutaneous emphysema occurs in the thoracic region secondary to pneumothorax or iatrogenic from chest tube/pigtail placement [2]. Spontaneous thoracic subcutaneous emphysema can also occur under increased intra-alveolar pressure, such as asthma, cough, and mechanical ventilation [3]. Sometimes, the air entrapment can extend to the cervical area and become increasingly large, leading to compression of the trachea and an airway emergency. Pelvic fractures can cause extensive subcutaneous emphysema, but this is typically in cases of injury to intrapelvic organs [4].

Retroperitoneal subcutaneous emphysema is a rare occurrence, most often iatrogenic following colonoscopy or abdominal surgery. It has been reported that approximately 0.016-5% of patients who underwent diagnostic colonoscopy developed subcutaneous emphysema [5]. Most of the case reports concerning abdominal subcutaneous emphysema come from developed nations where diverticular disease is prominent [6,7]. Generally, subcutaneous emphysema is localized to the abdominal region where colonic perforation occurs, as demonstrated in Kuhn’s case series, with rare extension to other areas of the body [8]. Colonic perforations have been reported to present with thigh or hip pain, which, in the setting of a fall, can be concerning for potential fractures. In these cases of colonic perforation, subcutaneous emphysema may be present; however, it is typically localized and not widespread [9]. Literature discussing mediastinal emphysema due to acute colon perforation is limited, with only 20 cases reported in the English language [4]. It is very rare for subcutaneous emphysema to extend from the abdominal region into the thoracic and cervical regions, mimicking a pneumothorax, or to the lower extremities, mimicking a pelvic or femur fracture in a trauma setting. To our knowledge, this is the first report of a spontaneous colonic perforation with such extensive subcutaneous emphysema that it mimicked a traumatic injury.

Diagnosis of subcutaneous emphysema is made via clinical exam findings, with confirmation through radiographic imaging, specifically CT scans. It is important to determine the cause of the subcutaneous emphysema to aid in treatment. Early diagnosis is extremely important in cases of acute perforated colon to allow prompt intervention. Generally, it is straightforward to diagnose pneumoperitoneum from a perforated colon in a classic presentation where a patient presents with acute abdominal pain, nausea, vomiting, and peritoneal signs [6]. However, challenges arise in cases with atypical presentations, such as this patient who presented after a fall with a presumed traumatic injury.

Since subcutaneous emphysema is typically a direct result of an underlying pathology, management is aimed at treating the underlying cause or precipitating factor. Typically, in mild cases, subcutaneous emphysema resolves in approximately 10 days without intervention if the source is controlled. High-concentration oxygen therapy may also aid in resolution, especially in patients with concomitant pneumothorax and/or pneumomediastinum. While it is rare, if the patient were to develop airway impingement or cardiovascular compromise due to extensive subcutaneous emphysema, infraclavicular incisions can be made bilaterally to help reduce the subcutaneous emphysema [1].

## Conclusions

Acute subcutaneous emphysema in the cervical, thoracic, retroperitoneal, and lower extremity regions is a rare presentation associated with acute perforated colon. Due to its rarity, alternative causes of subcutaneous emphysema are more likely to be initially presumed and evaluated. In this case, the patient was found on the ground and presented as hypotensive with swelling in his pelvis and thigh. Initially, his subcutaneous emphysema and hypotension were presumed to be traumatic. It was not until he underwent a CT scan that trauma was excluded and the final diagnosis was identified. Given the importance of early diagnosis and treatment of colonic perforation, this is an important case report identifying colonic perforation as a cause, albeit rare, of more diffuse subcutaneous emphysema.
